# Beyond microbial carbon use efficiency

**DOI:** 10.1093/nsr/nwae059

**Published:** 2024-02-07

**Authors:** Ke-Qing Xiao, Chao Liang, Zimeng Wang, Jingjing Peng, Yao Zhao, Ming Zhang, Mingyu Zhao, Shuiqing Chen, Yong-Guan Zhu, Caroline L Peacock

**Affiliations:** State Key Lab of Urban and Regional Ecology, Research Center for Eco-Environmental Sciences, Chinese Academy of Sciences, China; University of Chinese Academy of Sciences, China; Institute of Applied Ecology, Chinese Academy of Sciences, China; Department of Environmental Science and Engineering, Fudan University, China; College of Resources and Environmental Sciences, China Agricultural University, China; State Key Laboratory of Environmental Criteria and Risk Assessment, Chinese Research Academy of Environmental Sciences, China; State Key Laboratory of Agricultural Microbiology, College of Resources and Environment, Huazhong Agricultural University, China; Key Laboratory of Cenozoic Geology and Environment, Institute of Geology and Geophysics, Chinese Academy of Sciences, China; State Key Lab of Urban and Regional Ecology, Research Center for Eco-Environmental Sciences, Chinese Academy of Sciences, China; State Key Lab of Urban and Regional Ecology, Research Center for Eco-Environmental Sciences, Chinese Academy of Sciences, China; School of Earth and Environment, University of Leeds, UK

Microbial carbon use efficiency (CUE) is defined as the proportion of microbial biomass growth C versus substrate C uptake, and thus provides a useful measure of microbially driven accumulation and loss of soil organic carbon (SOC) [1]. In a recent study published in *Nature* [2], the authors used a data-driven machine-learning approach to conclude that CUE promotes global SOC storage based on a positive correlation between CUE and SOC content and that, based on sensitivity analysis, CUE is at least four times as important as six other evaluated factors, namely plant C inputs, C input allocation, non-microbial C transfer, substrate decomposability, environmental modifications and vertical transport. We agree with the authors that consensus in the scientific community about the relationship between CUE and SOC is important, and that increasingly used big data methods offer an opportunity to synthesize and potentially generate new insights from multiple data aggregation. We argue, however, that their study excludes important data sets and lacks mechanistic consideration of the complexities of SOC formation, such that their conclusions need to be clarified.

We posit that stabilization matters more than production (CUE) for SOC formation. The accumulation and persistence of SOC are affected by multiple factors, including biological, chemical and physical processes [3–5]. Microbial use of carbon represents the very primary stage of SOC formation (Fig. 1). When evaluating SOC storage, it is imperative to recognize that the stabilization process can matter more—a facet that was largely overlooked within the

**Figure 1.**
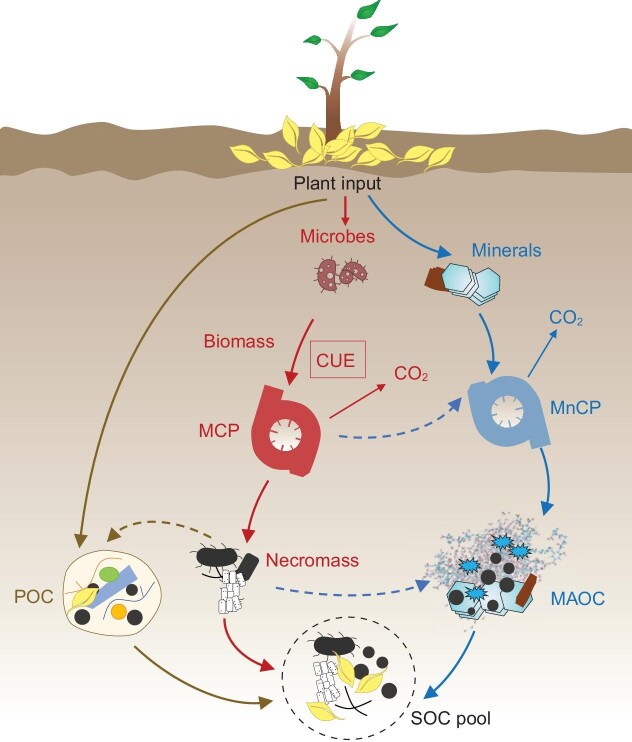
Conceptual diagram of microbial carbon use efficiency (CUE) and the stabilization mechanisms of soil organic carbon (SOC). MCP, microbial carbon pump; MnCP, mineral carbon pump; MAOC, mineral-associated organic carbon; POC, particulate organic carbon.

structure of the model by Tao *et al.* [2]. Microbial necromass may possess enhanced stability against decomposition (the microbial carbon pump) [6], but research also increasingly suggests that the production of microbial biomass and consequently necromass lead to a set of

organic compounds that are themselves stabilized against decomposition through a variety of chemical and physical processes (e.g. high activation energies for further decomposition and/or physico-chemical protection with mineral matrices) [3,4,7,8] (Fig. 1). For example, it is found that necromass accumulation is not solely dependent on CUE, but is strongly dependent on mechanisms preserving C components, most notably soil mineral content, with necromass accumulation occurring in soils with high clay-sized fraction [9]. In other work, CUE is found to be negatively correlated with persistent mineral-associated SOC, suggesting that necromass production is not the primary driver of SOC persistence [7]. In this work stimulation of microbial growth by high-quality litter enhances SOC decomposition, offsetting the positive effect of litter quality on SOC stabilization [7]. As such, CUE and SOC are decoupled rather than coupled in some environments [9,10]. This decoupling is also reflected in Extended Data Fig. 5c from Tao *et al.* [2], where there is no significant correlation between CUE and SOC in soil of >100 cm.

Meanwhile, it should be cautioned that correlation does not equal causation. In Tao *et al.* [2], model-derived CUE is an emergent property of the whole system from SOC profiles, and it is therefore not surprising that the calculated CUE is correlated with SOC (as in their Fig. 2b). Some important factors such as temperature have not been parameterized properly in the microbial model, so the conclusion that temperature does not have a big impact on SOC through the sensitivity analysis of this model becomes doubtful. A microbial model was used by the authors to examine the CUE–SOC relationship, yet the results (their Extended Data Fig. 4) clearly show that CUE–SOC correlation depends on the parameter chosen and can be either positively or negatively related. Even though a positive relationship between CUE and SOC may exist, we urge that more sophisticated empirical measurements should be done before a globally causal link between CUE and SOC can be established.

We also point out that data selection is critical for correlation results and biased analysis can lead to uncertainty and even misleading results. We argue that their meta-analysis needs more data in tropical and arid regions as well as clay soils (their Supplementary Fig. S4), while we posit that results based on 132 measurements are somewhat premature for a global assessment. Actually, the correlation between CUE and SOC for the data of 132 measurements is very weak (*R*^2^ = 0.11), and the correlation between CUE and log (SOC) is even weaker (*R*^2^ = 0.07) (Fig. 2). This strongly suggests that, while CUE and SOC may be related, CUE does not play a major role in determining SOC. We performed random forest analyses using data in Supplementary Table S1 from Tao *et al.* [2] and found that microbial biomass rather than CUE is among the most important predictors of SOC (Fig. 3). Moreover, the authors state that their results agree with findings from a landscape-scale pattern across the UK [11]. Whilst the data from that study (168 measurements) are not included in the 132 measurements for meta-analysis by Tao *et al.* [2] in their Fig. 2a, that study clearly states that soil pH is an important factor and the ‘CUE–SOC relationship broke down below the threshold pH (6.2)’ (Fig. 2a from Malik *et al.* [11]).

**Figure 2.**
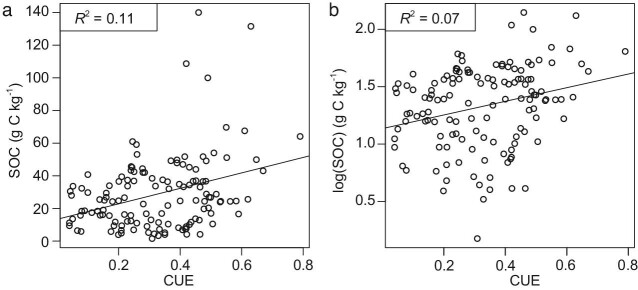
The correlation between CUE and SOC for the data of 132 measurements. (a) Correlation between CUE and SOC; (b) correlation between CUE and log (SOC). Public raw data from Supplementary Table S1 of Tao *et al.* [2]. CUE, microbial carbon use efficiency; SOC, soil organic carbon.

**Figure 3.**
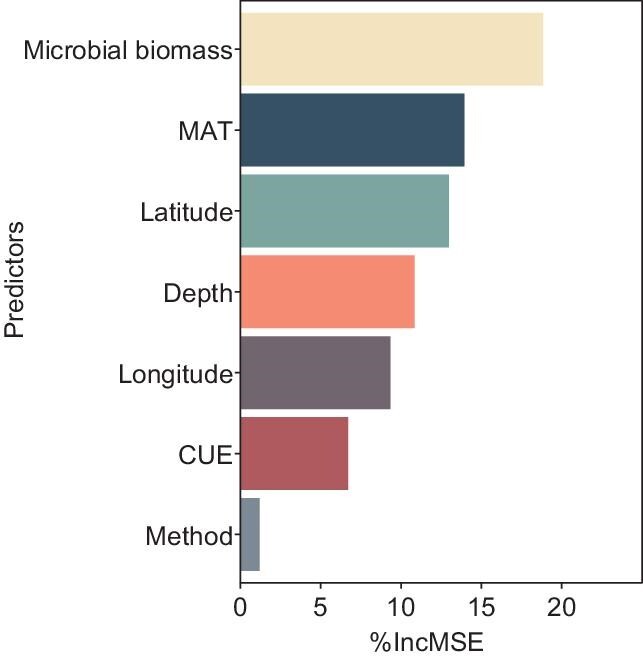
The relative importance of predictors in the random forest model predicting SOC contents. Public raw data from Supplementary Table S1 of Tao *et al.* [2]. MAT, mean annual temperature; CUE, microbial carbon use efficiency; Method, use of ^13^C or ^18^O for CUE measurement.

Overall, we argue that, while this study makes an important contribution towards our understanding of the links between CUE, microbial necromass and SOC persistence, it is premature to establish a globally robust causal relationship between CUE and SOC. We caution inferring mechanisms or causality from large data sets [12,13]. We posit that the analysis and conclusion would benefit from more consideration of mechanistic processes in SOC formation and caution when dealing with big data. While the strides made in data science have undoubtedly propelled our understanding in many fields, including soil science, we must exercise caution and not oversimplify intricate systems.

## Data Availability

All data are public.
